# The Principles and Practice of Dermatology

**Published:** 1907-10

**Authors:** 


**The Principles and Practice of Dermatology,** by **William Allen Pusey,** A.M., M.D., Professor of Dermatology in the University of Illinois. Octavo, pp. 1021. Illustrated. New York and London: D. Appleton & Company. 1907. (Price, $6.00.)

The author of this book was previously known as having collaborated with Eugene Wilson Caldwell in a work entitled "The Röntgen Rays in Therapeutics and Diagnosis," in which Pusey limits his subject matter to the treatment of skin diseases by the *x*-ray. In this particular he has been of great service to the profession at large. His former achievements will give to his present work additional importance.

The black and white illustrations are unusually comprehensive and are uniformly of a high order. They constitute an improvement in depicting conditions, thereby assisting to a better understanding of the description in the text itself. The author does not content himself with purely original pictures, but freely borrows from others when this will in anyway help to enforce his various points.

This work may be regarded as covering the entire field of dermatology and is entitled to a high place in scientific medicine. One of the features consists of combining the results of the rapid strides made in different countries in recent years; this, however, does not interfere with the strong individuality asserted in these pages.

Under the caption of secondary lesions, the author describes the special condition defined by most dermatologists as "lichenification," and by Jameison as "Leathery induration," improving on their description and enlarging thereon in a manner admirable and helpful to the student.

The proper practice of dermatology is clearly defined and in this connection the etiology, as well as the constant results of discovery are intelligently set forth. The anatomy of the skin and its appendages is thoroughly considered and fully illustrated. A great number of the illustrations are original. In dealing with general symptomology old truths are dwelt upon with a facility and force which renders them almost new. The system of classification adopted is like that of Hebra, Crocker and others but, best of all, are the modifications thereof by the author. These lead to a better understanding of disease, although it is to be regretted that some universal classification cannot be accepted and reduced to a definite system.

The description of the exanthemata occupies 28 pages; the author's suggestions are carefully worded and carry conviction with them. His descriptions and illustrations of these forms of acute infectious diseases are such as have not heretofore been found in works on dermatology. He takes the attitude that dermatologists should be more often consulted in the diagnosis and treatment of this class of diseases.

A special portion of the work is devoted to epithelioma. It appears, at first somewhat out of proportion to the rest of the text, but when it is considered that no skin condition leads to so many errors in diagnosis, especially in its earlier developments, it is none too fully treated. The condition is so intelligently described and so fully illustrated as to call for the highest praise from those whom it is especially intended to benefit. Too often the general practitioner knows but little of the true diagnosis of this condition, which is demonstrated by the fact that so many cases come before the dermatologists unaccompanied by any previous diagnosis. Certainly there should be more general intelligence with reference to maladies of the skin that are easy to conquer in their inceptive stage, but became incurable after the condition has been allowed to develop to a certain degree.

It would be useless to attempt to discuss those portions of the work referring to individual diseases. It is sufficient to say that the author's standard is high and that his conclusions are quite up to date. The aim of the book is to make more easy the recognition of skin diseases on the part of the general practitioner, and contains a comprehensive resume of symptomatology, pathology and treatment. Although condensed, the author's style is clear and nothing important seems to have been omitted.

Dr. Pusey is to be heartily complimented on the thoroughness with which he has completed his task. His book will fill a hiatus and will prove of very great value to dermatology. It is sure to become a handbook with the profession, and is quite certain to be regarded as indispensable to all who are engaged in dermatological research and practice.

